# Citrullinated peptides of peptidyl arginine deiminase 4 as major B-cell epitopes in patients with rheumatoid arthritis

**DOI:** 10.3389/fimmu.2025.1640291

**Published:** 2025-09-02

**Authors:** Mathilde Giassi, Eric Toussirot, Nathalie C. Lambert, Jean Roudier, Isabelle Auger

**Affiliations:** ^1^ Institut National de la Santé et de la Recherche Médicale (INSERM) Unité Mixte de Recherche (UMRs) 1097, Aix Marseille Université, Marseille, France; ^2^ Institut National de la Santé et de la Recherche Médicale (INSERM), Centre d’Investigation Clinique (CIC)-1431, Centre Investigation Clinique, Centre Hospitalier Universitaire (CHU) Besançon, Besançon, France

**Keywords:** rheumatoid arthritis, antibodies to citrullinated proteins, peptidyl arginine deiminase, peptide arrays, diagnosis

## Abstract

**Objective:**

To identify new autoantibodies in rheumatoid arthritis (RA) patients’ sera.

**Methods:**

We tested serum samples from 55 patients with RA and 25 controls on arrays containing 188 peptides from the alpha and beta chains of fibrinogen, vimentin, histon 4, enolase, proteoglycan, filaggrin, collagen, and human peptidyl arginine deiminase 4 (hPAD4). To confirm the validity of our peptide array detection, we tested serum samples from 50 patients with RA and 42 controls on purified peptides by luminescent enzyme-linked immunosorbent assay (ELISA).

**Results:**

We found citrullinated peptides from hPAD4 that were recognized almost uniquely by sera from patients with RA on peptide arrays and ELISA. Peptide P22/60 from hPAD4 is a better RA diagnostic tool than the major classical citrullinated B-cell epitopes from histon 4, proteoglycan, alpha fibrinogen, and enolase.

**Conclusion:**

We identified citrullinated peptides from hPAD4 as RA-specific autoantigens.

## Introduction

The major immunological event in rheumatoid arthritis (RA) is the development of autoantibodies directed against citrullinated proteins (proteins in which arginine residues have been converted into citrullines by peptidyl arginine deiminases). Anti-citrullinated protein autoantibodies (ACPAs) are highly specific and sensitive markers of RA, making them a part of its definition (1). Furthermore, they can be detected several years before the onset of clinical symptoms (2).

Human PAD4 (hPAD4), an enzyme that binds many different proteins and citrullinates them, is also an early target in RA (3, 4). Autoimmunity to hPAD4 precedes the clinical onset of RA (5). Moreover, autoantibodies to hPAD4 are present during the preclinical phase of RA and associated with ACPA in a subset of patients (6). Finally, ACPA immunity is associated with antibodies and T-cell responses to hPAD4 in RA patients (7).

ACPAs are detected by commercial ELISA kits containing synthetic cyclic citrullinated peptides (anti-CCP); nevertheless, they do not provide information about the peptides that are being recognized by these antibodies.

Here, we have developed a peptide array to identify new autoantibody signatures in patients with RA. This new and more sensitive screening system contains known citrullinated peptides that are major B-cell epitopes, as well as new citrullinated peptides from hPAD4.

## Patients and methods

### RA patients’ and controls’ sera

We studied 105 patients with RA and 31 with psoriatic arthritis (PsA) from the rheumatology units in Marseille and Besançon. RA patients fulfilled the 2010 ACR/EULAR criteria (1); 28% were newly diagnosed, while 72% were already receiving treatment at baseline. Psoriatic arthritis patients fulfilled the CASPAR criteria (8). We studied 36 healthy subjects from collection number DC-2008-327. Baseline characteristics of the patients and controls are presented in **Tables 1**, **2**.

**Table 1:** Subjects tested for the peptide array.

	RA patients	PsA patients	Healthy subjects
Number	55	13	12
Sex	35F	10F	8F
Disease duration (years)	1	3	
ACPA+	55/55	0/13	0/12
RF+	36/44	NT	NT
SE+	30/50	4/13	6/12
Treatment	16 none	4 none	
	10 NSAID		
	24 MTX	1 MTX	
	5 biotherapies	8 biotherapies	

RA, rheumatoid arthritis; PsA, psoriatic arthritis; F, female; ACPA, autoantibodies to citrullinated proteins; RF, rheumatoid factor; hPAD4, human peptidyl arginine deiminase 4; SE, shared epitope; MTX, methotrexate; NSAID, non-steroidal anti-inflammatory drug; NT, not tested.

**Table 2:** Subjects tested for ELISA.

	RA patients	PsA patients	Healthy subjects
Number	50	18	24
Sex	37F	13F	12F
Disease duration (years)	3	3.5	
ACPA+	50/50	4/18	0/24
RF+	14/19	NT	NT
SE+	28/46	4/18	9/24
Treatment	13 none	5 none	
	7 MTX	2 MTX	
	30 biotherapies	11 biotherapies	

RA, rheumatoid arthritis; PsA, psoriatic arthritis; F, female; ACPA, autoantibodies to citrullinated proteins; RF, rheumatoid factor; hPAD4, human peptidyl arginine deiminase 4; SE, shared epitope; MTX, methotrexate; NT, not tested.

### Study approval

All experimental protocols were approved by the Comité de Protection des Personnes, Sud-Méditerranée II, Ministère de l’Enseignement Supérieur et de la Recherche. Samples from patients and healthy subjects were collected under the collection number DC-2008-327. All participants gave informed consent.

### Peptide array

We tested 188 15-mers from the alpha and beta chains of fibrinogen, vimentin, histon 4, enolase, proteoglycan, filaggrin, collagen, and hPAD4. Peptides are listed in **Supplementary Table 1**. Citrulline was indicated by a Z. Peptides were synthesized and covalently immobilized on the array in triplicate (JPT Peptide Technologies, Berlin, Germany). Sera were diluted to 1/200 and incubated on peptide arrays. After washing, bound antibodies were detected using fluorescently labeled secondary antibodies (goat anti-human IgG, DyLight 650, Thermo Fisher Scientific, Illkirch-Graffenstaden, France). After washing and drying, the array was scanned at 635 nm (Axon GenePix Scanner 4300 SL50, JPT Peptide Technologies, Berlin, Germany). Images were quantified using the spot-recognition software GenePix (Molecular Devices, San Jose, USA). For each spot, mean signal intensity was extracted (between 0 and 65,535 arbitrary units). Mean background signal intensity was obtained for every peptide using all the sera from controls. A positive serum was defined by a ratio (mean test signal/mean background signal) higher than 3.

### Synthetic peptides for ELISA

15-mer peptides (ProteoGenix, Schiltigheim, France) were synthesized using the solid-phase system and purified (>70%).

### Luminescent ELISA

We have developed a luminescent method that is approximately 100 times more sensitive than a colorimetric ELISA. Briefly, plates were coated with 5 μg/well of peptide diluted in phosphate buffered saline (PBS), pH 7.4. Plates were blocked with PBS containing 2% bovine serum albumin (BSA). Sera diluted to 1:150 were incubated. After washing with PBS, peroxidase-conjugated anti-human IgG (Sigma, France) was added. After washing with PBS, a luminescent substrate (Sigma Aldrich, France) was added. After 5 min, light emission was detected using BioTek Gen5 (Agilent Technologies, France). Background signal was obtained for every peptide using all the sera from controls. A positive serum was defined as a signal higher than twice the mean background signal.

### Statistical analysis

The sample size calculator (Calculator.net) was used to determine the minimum number of participants required for each antibody detection method. We found that at least 69 participants were needed to estimate a proportion of 50% with a margin of error of 10% and a confidence level of 90%. Using two different methods across two separate cohorts allowed us to effectively identify key B-cell epitopes associated with RA. Comparisons between groups were performed using the Mann–Whitney tests and receiver operating characteristic (ROC) curve (GraphPad Prism version 10 software).

## Results

### Autoantibody detection by peptide arrays

Sera from 55 patients with RA (with 1 year of disease duration) and 25 controls were used to probe the array containing 188 peptides from the alpha and beta chains of fibrinogen, vimentin, histon 4, enolase, proteoglycan, filaggrin, collagen, and hPAD4. The presence of autoantibodies bound to each peptide was detected with a fluorescently labeled anti-human IgG antibody. Mean background signal intensity was obtained for every peptide using all the sera from controls. A positive serum was defined by a ratio (mean test signal/mean background signal) higher than 3.

**Figure 1:**
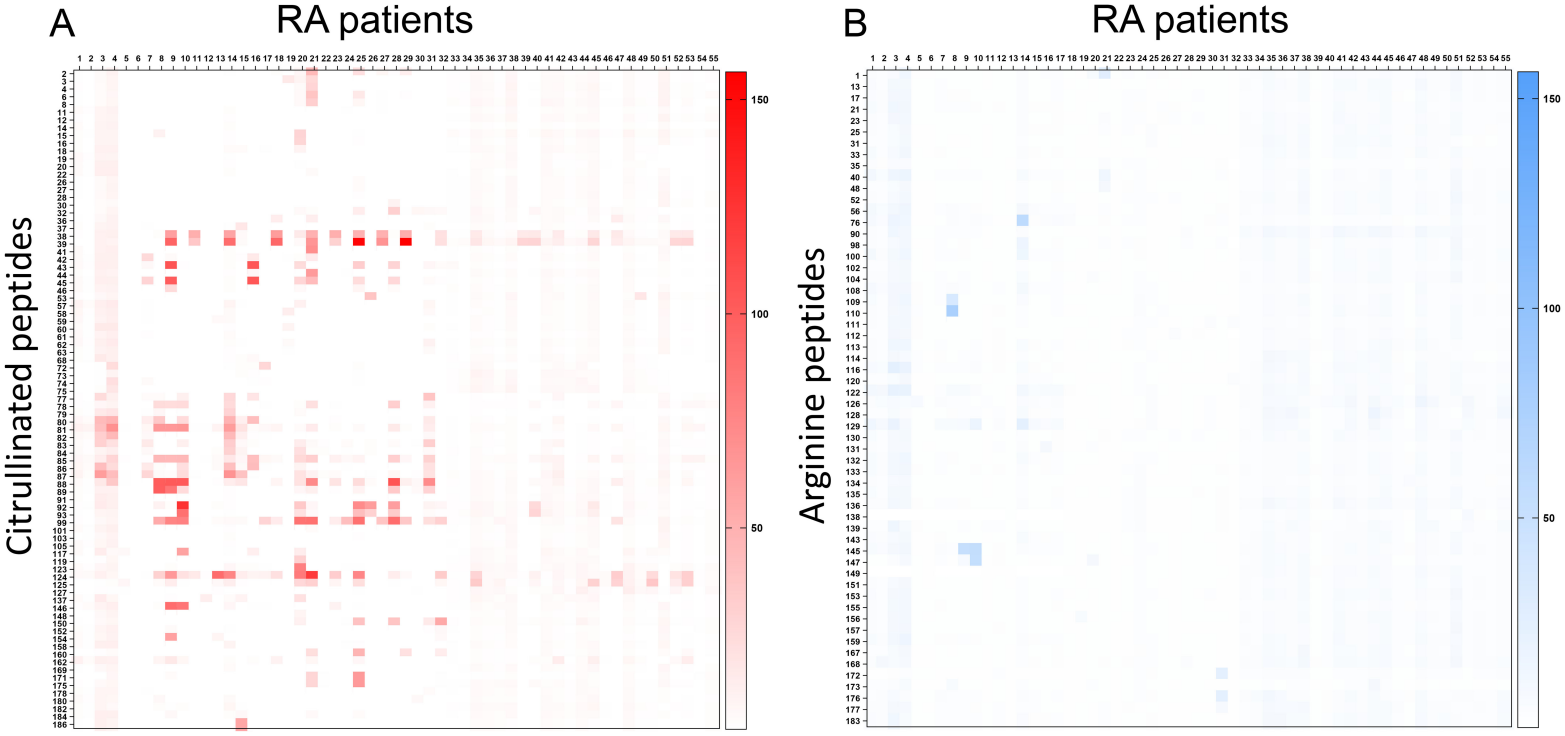
Detection of B-cell epitopes in patients with RA by peptide arrays. A heatmap diagram was computed with ratios (mean test signal/mean background signal) in a color-coded manner from white (no binding) to red (strong binding) for citrullinated peptides **(A)** and from white (no binding) to blue (strong binding) for arginine peptides **(B)**. The X-axis represents patients with RA, and the Y-axis represents peptides recognized by patients with RA and any control serum.

Most peptides were recognized by the sera of patients with RA under their citrullinated form (**Figure 1**). Among citrullinated peptides recognized by more than 50% of RA patients and 0% of controls, 14 were known B-cell epitopes in RA: these were peptides from histon 4, proteoglycan, alpha fibrinogen, and enolase (**Table 3**).

**Table 3:** Detection of major B-cell epitopes by peptide arrays in patients with RA.

Index	Sequence	Protein	Peptide name	Arginine (R) or citrulline (CIT)	Average ratios for RA patients	% of positive RA patients	Average ratios for controls	% of positive controls
**124**	**VRVFQATZGKLSSKC**	**PAD4**	**22/60**	**CIT**	**18**	**82**	**1**	**0**
85	AIRRLAZZGGVKRIS	histon	his 39–40 plus	CIT	11	80	1	0
99	LRVTZGSRAPVSRAQ	proteoglycan	P46-60	CIT	17	78	1	0
88	AIZZLAZZGGVKRIS	histon	his 39-40	CIT	16	78	1	0
81	AIZRLARZGGVKRIS	histon	his 39-40	CIT	12	76	1	0
38	RGHAKSRPVZGIHTS	alpha fibrinogen	a621-635	CIT	14	71	1	0
87	AIZZLAZRGGVKRIS	histon	his 39-40	CIT	9	71	1	0
39	ZGHAKSZPVZGIHTS	alpha fibrinogen	a621-635	CIT	21	69	1	0
92	IHAREIFDSZGNPTV	enolase	CEP 6-20	CIT	11	67	1	0
**125**	**VZVFQATZGKLSSKC**	**PAD4**	**22/61**	**CIT**	**8**	**67**	**1**	**0**
89	AIZZLAZZGGVKZIS	histon	his 39-40	CIT	9	60	1	0
83	AIRZLAZRGGVKRIS	histon	his 39-40	CIT	6	60	1	0
**123**	**VZVFQATRGKLSSKC**	**PAD4**	**22/59**	**CIT**	**5**	**58**	**1**	**0**
86	AIRRLAZRGGVKZIS	histon	his 39-40	CIT	7	56	1	0
122	VRVFQATRGKLSSKC	PAD4	22	R	4	56	1	0
78	AIRRLARZGGVKRIS	histon	his 39-40	CIT	6	55	1	0
**150**	**IKZVMGPDFGYVTZG**	**PAD4**	**39**	**CIT**	**6**	**53**	**1**	**0**
2	GGGVZGPRVVERHQS	alpha fibrinogen	a31-45h	CIT	5	53	1	0
84	AIRZLARRGGVKZIS	histon	his 39-40	CIT	4	53	1	0

Citrulline was indicated by a Z.

The bolded text indicates the citrullinated peptides of hPAD4.

Four were new B-cell epitopes from hPAD4 (**Table 3**): peptide P22/60 (VRVFQATZGKLSSKC) was recognized by 82% of RA patients with an average ratio of 18 (± 26 SD), peptide P22/61 (VZVFQATZGKLSSKC) was recognized by 67% of RA patients with an average ratio of 8 ( ± 10 SD), peptide P22/59 (VZVFQATRGKLSSKC) was recognized by 58% of RA patients with an average ratio of 5 ( ± 10 SD), and peptide P39 (IKZVMGPDFGYVTZG) was recognized by 53% of RA patients with an average ratio of 6 ( ± 10 SD).

### Autoantibody validation by ELISA

**Figure 2:**
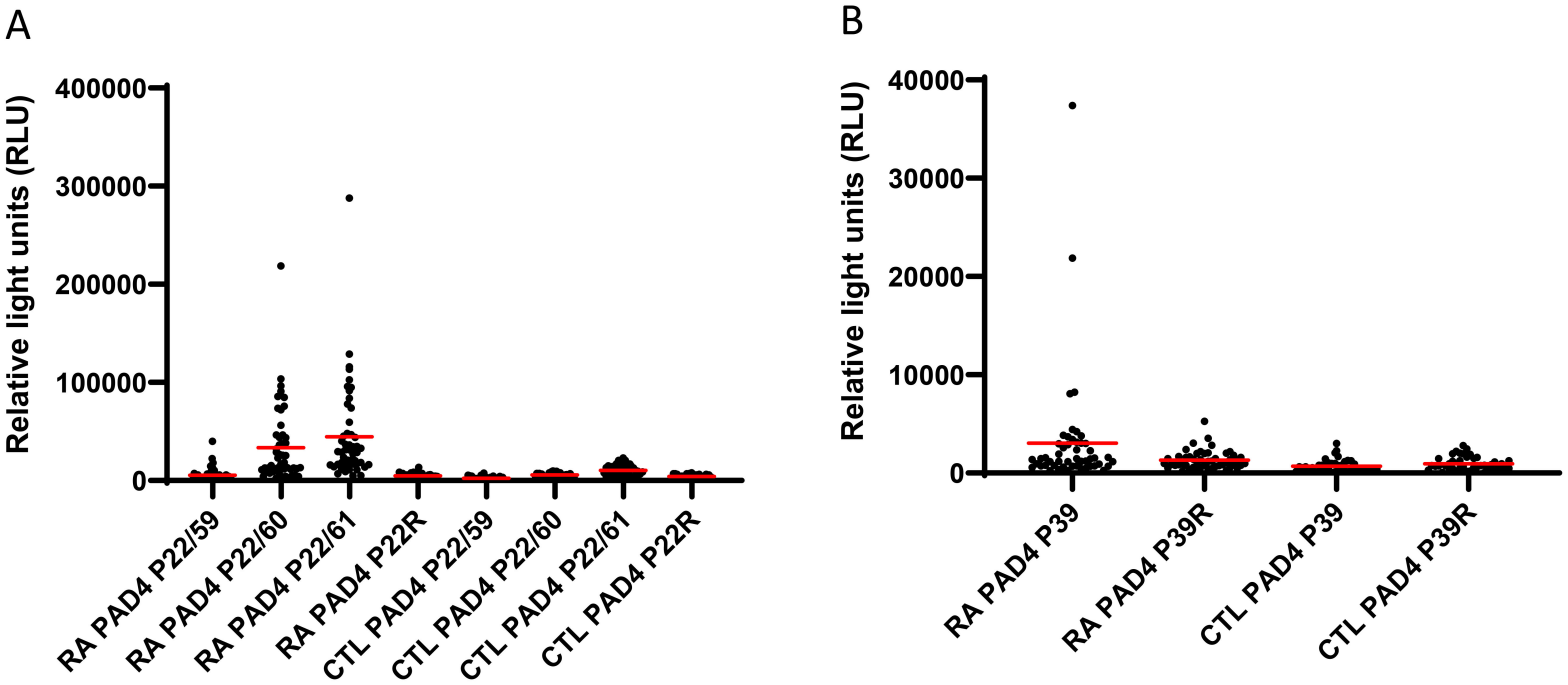
Detection of autoantibodies to peptides from hPAD4 by ELISA. Sera from patients with RA and 42 controls were used to probe plates containing citrullinated peptides P22/59, P22/60, P22/61 **(A)**, and P39 **(B)** from hPAD4. Their arginine-substituted variants P22R and P39R were also tested. The presence of autoantibodies bound to each peptide was detected by an anti-human IgG antibody. Background signal was obtained for every peptide using all the sera from controls. A positive serum was defined as a signal higher than twice the mean background signal. Means are indicated in red. Results are presented in two separate graphs due to scale differences in relative light unit results between peptides.

Sera from 50 patients with RA (with a disease duration of 3 years) and 42 controls were used to probe plates containing citrullinated peptides P22/59, P22/60, P22/61, and P39 from hPAD4 and their arginine-substituted variants. The presence of autoantibodies bound to each peptide was detected by an anti-human IgG antibody (**Figure 2**).

Peptide P22/60 was recognized by 76% of RA patients with an average signal of 33,589 ( ± 38,780 SD) versus 0 of 42 (0%) controls with an average signal of 5,565 ( ± 2,413 SD) (Mann–Whitney test, RA patients versus controls: *p* < 0.0001; **Figure 2A**).

Peptide P22/61 was recognized by 62% of RA patients with an average signal of 44,484 ( ± 47,956 SD) versus 2% of controls with an average signal of 10,314 ( ± 5,472 SD) (Mann–Whitney test, RA patients versus controls: *p* < 0.0001; **Figure 2A**).

Peptide P22/59 was recognized by 34% of RA patients with an average signal of 5,318 ( ± 6,744 SD) versus 9% of controls with an average signal of 2,276 ( ± 1,589 SD) (Mann–Whitney test, RA patients versus controls: *p* = 0.0012; **Figure 2A**).

Peptide P39 was recognized by 50% of RA patients with an average signal of 3,030 ( ± 5,958 SD) versus 12% of controls with an average signal of 693 ( ± 658 SD) (Mann–Whitney test, RA patients versus controls: *p* < 0.0001; **Figure 2B**).

**Figure 3:**
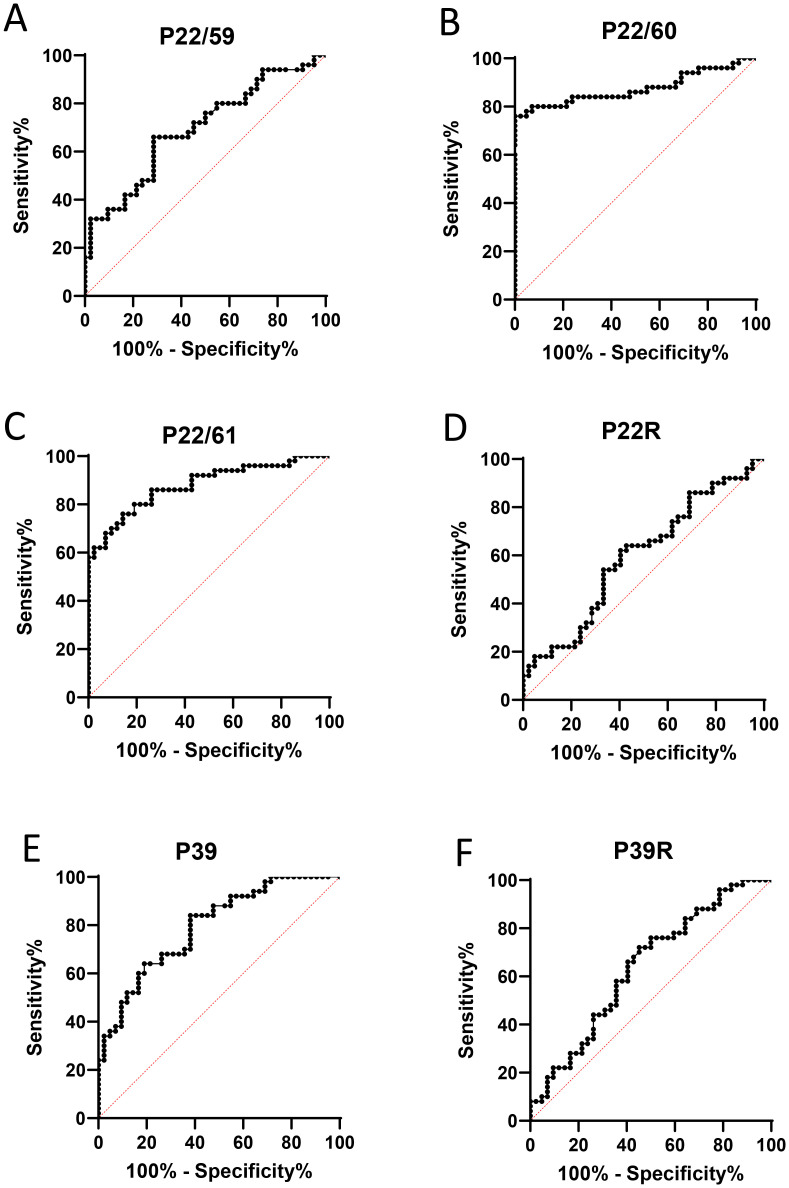
**(A–F)** Validation of citrullinated peptides from hPAD4 as RA-specific autoantigens. IgG responses to peptides from hPAD4, obtained with the sera from 50 patients with RA and 42 controls in ELISA, were analyzed using an ROC curve.

Peptide P22/60 was the best predictor of RA with an area under the curve (AUC) of 0.875 for predicting RA (0.039 std. error, *p* < 0.0001), with 76% sensitivity and 100% specificity (**Figure 3**).

## Discussion

We have developed a new peptide array containing 78 arginine-containing peptides and their 110 citrulline-substituted variants to be able to analyze at the same time the presence of ACPAs and anti-hPAD4 antibodies in the sera from patients with RA.

We designed 103 15-mers (25 native, 78 citrullinated) from the alpha and beta chains of fibrinogen, vimentin, histon 4, enolase, proteoglycan, filaggrin, and collagen, all of which are known to be recognized by ACPAs (9–13). These peptides are centered on Cit-Gly motifs to optimize the binding of ACPAs (14). We also included 85 15-mers (53 native and 32 citrullinated) from hPAD4.

Autoantibodies from RA patients recognize peptides from many proteins, most of which are citrullinated. We identified major citrullinated B-cell epitopes from histon 4, proteoglycan, alpha fibrinogen, and enolase that have already been described.

We identified a new citrullinated autoantigen that is specific for patients with RA: peptide P22/60 from hPAD4. Its sensitivity is 76%, while its specificity is 100%.

This result could help reduce diagnostic delays by providing a specific biomarker to distinguish RA from PsA. Such differentiation is essential, as RA and PsA require different treatment strategies, and early, targeted intervention can significantly improve patient outcomes.

Our study has limitations. At first, the sample size is moderate, which may affect the generalizability of the findings. Indeed, the production of autoantibodies against citrullinated PAD4 peptides was tested on a group of 172 patients and controls. Secondly, the cohorts are geographically homogeneous, being based solely in France, which might limit the applicability of results to more diverse populations. Lastly, the study lacks longitudinal follow-up, preventing us from assessing the predictive value of the variables over time.

Future research involving larger, more diverse cohorts and longitudinal designs will help validate and expand our results. This will allow us to analyze how the production of these autoantibodies evolves over time, assess the effect of treatment on their production, and explore correlations with HLA-DRB1 genotypes. Finally, the longitudinal study will allow a comparison of the specificity, sensitivity, and early detection values of the citrullinated P22/60 peptide from hPAD4 with those of the cyclic citrullinated peptide used in the CCP2 kit. If the validation of the citrullinated P22/60 peptide from hPAD4 confirms its specificity, sensitivity, and early detection capabilities for RA, it could be incorporated into diagnostic kits.

Peptide P22/60 from hPAD4 is located in the N-terminal calcium-binding domain of hPAD4 (amino acids 211 to 225).

This might indicate that hPAD4 undergoes self-citrullination and is thus recognized by RA-specific anti-citrulline antibodies. Quantitative proteomics studies have shown that the arginine residues at positions 212 and 218 are preferential sites of self-citrullination (15).

The simultaneous presence of autoantibodies to hPAD4 and citrulline in most RA patients may indicate that the event, which initiates anti-citrulline immunization, involves a complex associating hPAD4 and a target protein undergoing citrullination.

**Figure 4:**
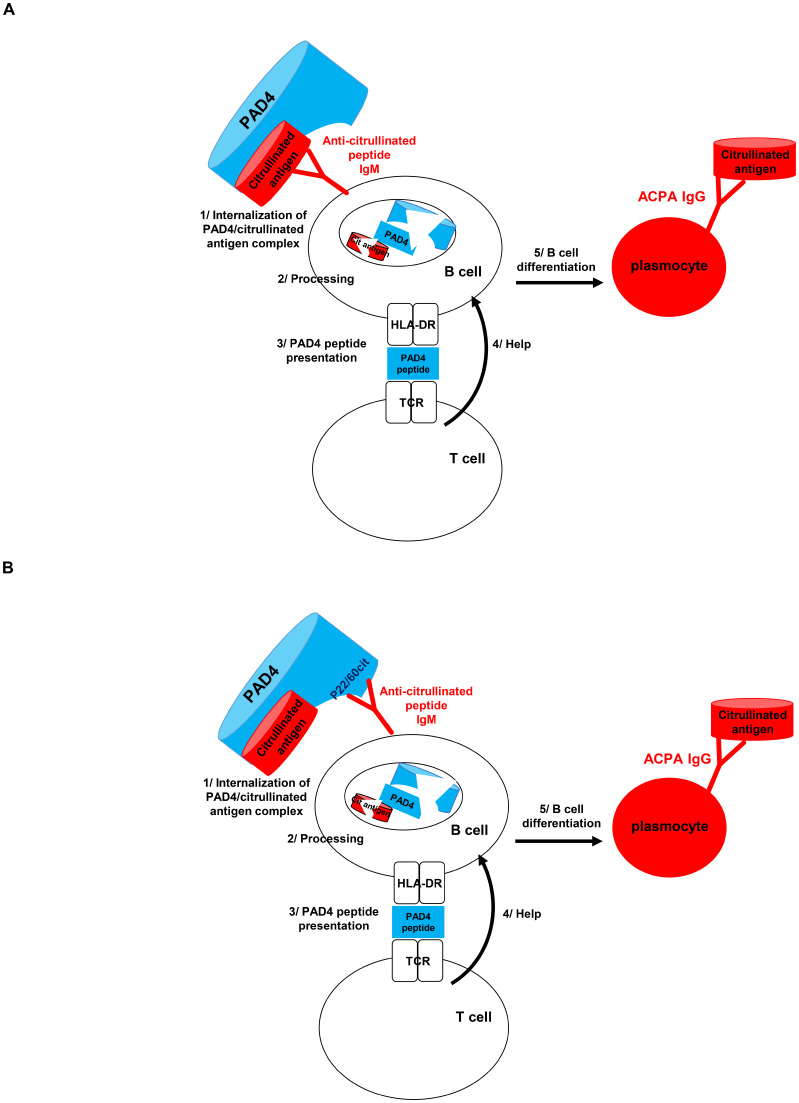
Hapten/carrier model. B cells recognizing proteins being bound and citrullinated by hPAD4 could process the hPAD4/citrullinated protein complexes and present peptides from hPAD4 to helper T cells **(A)**. B cells recognizing citrullinated peptides from hPAD4 could process the hPAD4/citrullinated protein complexes and present peptides from hPAD4 to helper T cells **(B)**.

In 2017, we proposed that T-cell recognition of hPAD4 provides help for the production of autoantibodies to citrullinated proteins, by a hapten carrier mechanism, in which the carrier is hPAD4 and the haptens are the multiple proteins being bound and citrullinated by hPAD4 (16). Indeed, citrullinated epitope-specific B lymphocytes could bind citrullinated epitopes complexed with hPAD4 (**Figure 4A**), process the hPAD4/citrullinated peptide complexes, and present peptides from hPAD4 processed from the complexes to helper T cells. Thus, B cells specific for citrullinated epitopes benefit from the help of hPAD4-specific helper T cells. From 2017 to 2022, we demonstrated this mechanism in a mouse model of RA and in patients with RA (7, 16–19). We recently demonstrated that hPAD4 tolerization can stop the carrier effect and decrease ACPA production (20).

Peptide P22/60 from hPAD4 may be used as a therapeutic target (patent no. 23508). This peptide may be involved in the internalization of hPAD4/citrullinated protein complexes by B cells in RA patients (**Figure 4B**). Blocking this process by eliminating B cells specific for peptide P22/60 could prevent the immune response to hPAD4 and thereby inhibit the production of ACPA. B cells specific for peptide P22/60 could be eliminated by natural killer cells or macrophages via antibody-dependent cell-mediated cytotoxicity or antibody-dependent cellular phagocytosis mechanisms.

Finally, the hapten carrier mechanism is expected to provide a selective advantage for B cells recognizing citrullinated peptides from hPAD4 to process the hPAD4/citrullinated peptide complexes and present peptides from hPAD4 to helper T cells. Thus, this model predicts that the first citrullinated epitope recognized by autoantibodies in RA should be located on hPAD4. This is consistent with what is observed here with peptide P22/60 from hPAD4.

## Data Availability

The raw data supporting the conclusions of this article will be made available by the authors, without undue reservation.
