# Supplementary material for: Citrullinated peptides of peptidyl arginine deiminase 4 as major B-cell epitopes in patients with rheumatoid arthritis
Source: Front Immunol. 2025 Sep 2;16:1640291. doi: 10.3389/fimmu.2025.1640291 (PMC12437702; doi:10.3389/fimmu.2025.1640291)
Supplement: Supplementary file 1 [file DataSheet1.pdf]

Supplementary table 1: Peptides in peptide arrays

Index	Sequence	Protein	peptide name	Arginine (R) or citrulline (CIT)
1	GGGVRGPRVVERHQS	alpha fibrinogen	a31-45h	R
2	GGGVZGPRVVERHQS	alpha fibrinogen	a31-45h	CIT
3	GGGVRGPZVVERHQS	alpha fibrinogen	a31-45h	CIT
4	GGGVRGPRVVEZHQS	alpha fibrinogen	a31-45h	CIT
5	GGGVZGPZVVERHQS	alpha fibrinogen	a31-45h	CIT
6	GGGVZGPRVVEZHQS	alpha fibrinogen	a31-45h	CIT
7	GGGVRGPZVVEZHQS	alpha fibrinogen	a31-45h	CIT
8	GGGVZGPZVVEZHQS	alpha fibrinogen	a31-45h	CIT
9	GPRVVERHQSACKDS	alpha fibrinogen	a36-50h	R
10	GPZVVERHQSACKDS	alpha fibrinogen	a36-50h	CIT
11	GPRVVEZHQSACKDS	alpha fibrinogen	a36-50h	CIT
12	GPZVVEZHQSACKDS	alpha fibrinogen	a36-50h	CIT
13	VDIDIKIRSCRGSCS	alpha fibrinogen	a171-185	R
14	VDIDIKIZSCRGSCS	alpha fibrinogen	a171-185	CIT
15	VDIDIKIRSCZGSCS	alpha fibrinogen	a171-185	CIT
16	VDIDIKIZSCZGSCS	alpha fibrinogen	a171-185	CIT
17	SCSRALAREVDLKDY	alpha fibrinogen	a183-197	R
18	SCSZALAREVDLKDY	alpha fibrinogen	a183-197	CIT
19	SCSRALAZEVDLKDY	alpha fibrinogen	a183-197	CIT
20	SCSZALAZEVDLKDY	alpha fibrinogen	a183-197	CIT
21	ERGSAGHWTSESSVS	alpha fibrinogen	a366-380	R
22	EZGSAGHWTSESSVS	alpha fibrinogen	a366-380	CIT
23	GTFEEVSGNVSPGTR	alpha fibrinogen	a411-425	R
24	GTFEEVSGNVSPGTZ	alpha fibrinogen	a411-425	CIT
25	SGIGTLDGFRHRHPD	alpha fibrinogen	a501-515	R
26	SGIGTLDGFZHRHPD	alpha fibrinogen	a501-515	CIT
27	SGIGTLDGFRHZHPD	alpha fibrinogen	a501-515	CIT
28	SGIGTLDGFZHZHPD	alpha fibrinogen	a501-515	CIT
29	SRGSESGIFTNTKES	alpha fibrinogen	a546-560	R
30	SZGSESGIFTNTKES	alpha fibrinogen	a546-560	CIT
31	SSHHPGIAEFPSRGK	alpha fibrinogen	a561-675	R
32	SSHHPGIAEFPSZGK	alpha fibrinogen	a561-675	CIT
33	SYNRGDSTFESKSYK	alpha fibrinogen	a588-602	R
34	SYNZGDSTFESKSYK	alpha fibrinogen	a588-602	CIT
35	RGHAKSRPVRGIHTS	alpha fibrinogen	a621-635	R
36	ZGHAKSRPVRGIHTS	alpha fibrinogen	a621-635	CIT
37	RGHAKSZPVRGIHTS	alpha fibrinogen	a621-635	CIT
38	RGHAKSRPVZGIHTS	alpha fibrinogen	a621-635	CIT
39	ZGHAKSZPVZGIHTS	alpha fibrinogen	a621-635	CIT
40	RPAPPPISGGGYRAR	beta fibrinogen	b60-74	R
41	ZPAPPPISGGGYRAR	beta fibrinogen	b60-74	CIT
42	RPAPPPISGGGYZAR	beta fibrinogen	b60-74	CIT
43	RPAPPPISGGGYRAZ	beta fibrinogen	b60-74	CIT
44	ZPAPPPISGGGYZAR	beta fibrinogen	b60-74	CIT
45	ZPAPPPISGGGYRAZ	beta fibrinogen	b60-74	CIT
46	RPAPPPISGGGYZAZ	beta fibrinogen	b60-74	CIT
47	ZPAPPPISGGGYZAZ	beta fibrinogen	b60-74	CIT
48	APPPISGGGYRARPA	beta fibrinogen	b62-76	R
49	APPPISGGGYZARPA	beta fibrinogen	b62-76	CIT
50	APPPISGGGYRAZPA	beta fibrinogen	b62-76	CIT
51	APPPISGGGYZAZPA	beta fibrinogen	b62-76	CIT
52	VIQNRQDGSVDFGRK	beta fibrinogen	b281-295	R
53	VIQNZQDGSVDFGZK	beta fibrinogen	b281-295	CIT
54	VVWMNWKGSWYSMRK	beta fibrinogen		R
55	VVWMNWKGSWYSMZK	beta fibrinogen		CIT
56	STRSVSSSSYRRMFG	vimentin	vim 2-16	R
57	STZSVSSSSYRRMFG	vimentin	vim 2-16	CIT
58	STRSVSSSSYZRMFG	vimentin	vim 2-16	CIT
59	STRSVSSSSYRZMFG	vimentin	vim 2-16	CIT
60	STZSVSSSSYZRMFG	vimentin	vim 2-16	CIT
61	STZSVSSSSYRZMFG	vimentin	vim 2-16	CIT
62	STRSVSSSSYZZMFG	vimentin	vim 2-16	CIT
63	STZSVSSSSYZZMFG	vimentin	vim 2-16	CIT
64	SAVRLRSSVPGVRLL	vimentin		R
65	SAVZLRSSVPGVRLL	vimentin		CIT
66	SAVRLZSSVPGVRLL	vimentin		CIT
67	SAVZLZSSVPGVRLL	vimentin		CIT
68	SAVZLZSSVPGVZLL	vimentin		CIT
69	GAKRHRKVLRDNIQG	histon	his 14-28	R
70	GAKZHRKVLRDNIQG	histon	his 14-28	CIT
71	GAKRHZKVLRDNIQG	histon	his 14-28	CIT
72	GAKRHRKVLZDNIQG	histon	his 14-28	CIT
73	GAKZHZKVLRDNIQG	histon	his 14-28	CIT
74	GAKZHRKVLZDNIQG	histon	his 14-28	CIT
75	GAKZHZKVLZDNIQG	histon	his 14-28	CIT
76	AIRRLARRGGVKRIS	histon	his 39-40	R
77	AIRRLAZRGGVKRIS	histon	his 39-40	CIT
78	AIRRLARZGGVKRIS	histon	his 39-40	CIT
79	AIRRLARRGGVKZIS	histon	his 39-40	CIT
80	AIZRLAZRGGVKRIS	histon	his 39-40	CIT
81	AIZRLARZGGVKRIS	histon	his 39-40	CIT
82	AIZRLARRGGVKZIS	histon	his 39-40	CIT
83	AIRZLAZRGGVKRIS	histon	his 39-40	CIT
84	AIRZLARRGGVKZIS	histon	his 39-40	CIT
85	AIRRLAZZGGVKRIS	histon	his 39-40 plus	CIT
86	AIRRLAZRGGVKZIS	histon	his 39-40	CIT
87	AIZZLAZRGGVKRIS	histon	his 39-40	CIT
88	AIZZLAZZGGVKRIS	histon	his 39-40	CIT
89	AIZZLAZZGGVKZIS	histon	his 39-40	CIT
90	IHAREIFDSRGNPTV	enolase	CEP 6-20	R
91	IHAZEIFDSRGNPTV	enolase	CEP 6-20	CIT
92	IHAREIFDSZGNPTV	enolase	CEP 6-20	CIT
93	IHAZEIFDSZGNPTV	enolase	CEP 6-20	CIT
94	TAKGLFRAAVPSGAS	enolase	CEP 26-40	R
95	TAKGLFZAAVPSGAS	enolase	CEP 26-40	CIT

96	MDMCSAGWLADRSVR	Proteoglycan	P49	R
97	MDMCSAGWLADZSVZ	proteoglycan	P49	CIT
98	LRVTRGSRAPVSRAQ	Proteoglycan	P46-60	R
99	LRVTZGSRAPVSRAQ	proteoglycan	P46-60	CIT
100	STRGRSRGRSGRSGS	filaggrin		R
101	STZGZSZGZSGZSGS	filaggrin		CIT
102	GARGLTGNPGVQGPE	collagen	568-582	R
103	GAZGLTGNPGVQGPE	collagen	568-582	CIT
104	MAQGTLIRVTPEQPT	PAD4		R
105	MAQGTLIZVTPEQPT	PAD4		CIT
106	LIRVTPEQPTHAVCV	PAD4		R
107	LIZVTPEQPTHAVCV	PAD4		CIT
108	PEQPTHAVCVLGTLT	PAD4		R
109	HAVCVLGTLTQLDIC	PAD4		R
110	LGTLTQLDICSSAPE	PAD4		R
111	SSAPEDCTSFSINAS	PAD4		R
112	VTLTMKVASGSTGDQ	PAD4		R
113	ALLYLTGVEISLCAD	PAD4		R
114	SLCADITRTGKVKPT	PAD4		R
115	SLCADITZTGKVKPT	PAD4		CIT
116	ITRTGKVKPTRAVKD	PAD4		R
117	ITZTGKVKPTZAVKD	PAD4		CIT
118	KVKPTRAVKDQRTWT	PAD4		R
119	KVKPTZAVKDQZTWT	PAD4		CIT
120	LHVARSEMDKVRVFQ	PAD4		R
121	LHVAZSEMDKVZVFQ	PAD4		CIT
122	VRVFQATRGKLSSKC	PAD4	22	R
123	VZVFQATRGKLSSKC	PAD4	22/59	CIT
124	VRVFQATZGKLSSKC	PAD4	22/60	CIT
125	VZVFQATZGKLSSKC	PAD4	22/61	CIT
126	ATRGKLSSKCSVVLG	PAD4		R
127	ATZGKLSSKCSVVLG	PAD4		CIT
128	LSSKCSVVLGPKWPS	PAD4		R
129	SVVLGPKWPSHYLMV	PAD4		R
130	PGGKHNMDFYVEALA	PAD4		R
131	VEALAFPDTDFPGLI	PAD4		R
132	FPGLITLTISLLDTS	PAD4		R
133	TLTISLLDTSNLELP	PAD4		R
134	LLDTSNLELPEAVVF	PAD4		R
135	NLELPEAVVFQDSVV	PAD4		R
136	FRVAPWIMTPNTQPP	PAD4		R
137	FZVAPWIMTPNTQPP	PAD4		CIT
138	KSVTTLAMKAKCKLT	PAD4		R
139	LAMKAKCKLTICPEE	PAD4		R
140	YIQAPHKTLPVVFDS	PAD4		R
141	HKTLPVVFDSPRNRG	PAD4		R
142	HKTLPVVFDSPZNZG	PAD4		CIT
143	VVFDSPRNRGLKEFP	PAD4		R
144	VVFDSPZNZGLKEFP	PAD4		CIT
145	PRNRGLKEFPIKRVM	PAD4		R
146	PZNZGLKEFPIKZVM	PAD4		CIT
147	LKEFPIKRVMGPDFG	PAD4		R
148	LKEFPIKZVMGPDFG	PAD4		CIT
149	IKRVMGPDFGYVTRG	PAD4		R
150	IKZVMGPDFGYVTZG	PAD4	39	CIT
151	GPDFGYVTRGPQTGG	PAD4		R
152	GPDFGYVTZGPQTGG	PAD4		CIT
153	YVTRGPQTGGISGLD	PAD4		R
154	YVTZGPQTGGISGLD	PAD4		CIT
155	PQTGGISGLDSFGNL	PAD4		R
156	ISGLDSFGNLEVSPP	PAD4		R
157	SFGNLEVSPPVTVRG	PAD4		R
158	SFGNLEVSPPVTVZG	PAD4		CIT
159	EVSPPVTVRGKEYPL	PAD4		R
160	EVSPPVTVZGKEYPL	PAD4		CIT
161	VTVRGKEYPLGRILF	PAD4		R
162	VTVZGKEYPLGZILF	PAD4		CIT
163	GRILFGDSCYPSNDS	PAD4		R
164	GZILFGDSCYPSNDS	PAD4		CIT
165	GDSCYPSNDSRQMHQ	PAD4		R
166	GDSCYPSNDSZQMHQ	PAD4		CIT
167	VQAPVKLYSDWLSVG	PAD4		R
168	HVDEFLSFVPAPDRK	PAD4		R
169	HVDEFLSFVPAPDZK	PAD4		CIT
170	APDRKGFRLLLASPR	PAD4		R
171	APDZKGFZLLLASPZ	PAD4		CIT
172	NEGHGEALLFEGIKK	PAD4		R
173	KKQQKIKNILSNKTL	PAD4		R
174	FVERCIDWNRELLKR	PAD4		R
175	FVEZCIDWNZELLKZ	PAD4		CIT
176	VLGKHLGIPKPFGPV	PAD4		R
177	PFGPVINGRCCLEEK	PAD4		R
178	PFGPVINGZCCLEEK	PAD4		CIT
179	QCTFINDFFTYHIRH	PAD4		R
180	QCTFINDFFTYHIZH	PAD4		CIT
181	NDFFTYHIRHGEVHC	PAD4		R
182	NDFFTYHIZHGEVHC	PAD4		CIT
183	YHIRHGEVHCGTNVR	PAD4		R
184	YHIZHGEVHCGTNVZ	PAD4		CIT
185	GEVHCGTNVRRKPFS	PAD4		R
186	GEVHCGTNVZZKPFS	PAD4		CIT
187	VRRKPFSFKWWNMVP	PAD4		R
188	VZZKPFSFKWWNMVP	PAD4		CIT

Z: citrulline
